# Planned improvement actions based on patient safety incident reports in Estonian hospitals: a document analysis

**DOI:** 10.1136/bmjoq-2022-002058

**Published:** 2023-05-15

**Authors:** Ere Uibu, Kaja Põlluste, Margus Lember, Karolin Toompere, Mari Kangasniemi

**Affiliations:** 1 Institute of Family Medicine and Public Health, University of Tartu, Tartu, Estonia; 2 Institute of Clinical Medicine, University of Tartu, Tartu, Estonia; 3 Department of Nursing Science, University of Turku, Turku, Finland

**Keywords:** patient safety, incident reporting, healthcare quality improvement

## Abstract

**Aim:**

Aim of this study was to describe and analyse associations of incidents and their improvement actions in hospital setting.

**Methods:**

It was a retrospective document analysis of incident reporting systems’ reports registered during 2018–2019 in two Estonian regional hospitals. Data were extracted, organised, quantified and analysed by statistical methods.

**Results:**

In total, 1973 incident reports were analysed. The most commonly reported incidents were related to patient violent or self-harming behaviour (n=587), followed by patient accidents (n=379), and 40% of all incidents were non-harm incidents (n=782). Improvement actions were documented in 83% (n=1643) of all the reports and they were focused on (1) direct patient care, (2) staff-related actions; (3) equipment and general protocols and (4) environment and organisational issues. Improvement actions were mostly associated with medication and transfusion treatment and targeted to staff. The second often associated improvement actions were related to patient accidents and were mostly focused on that particular patient’s further care. Improvement actions were mostly planned for incidents with moderate and mild harm, and for incidents involving children and adolescents.

**Conclusion:**

Patient safety incidents-related improvement actions need to be considered as a strategy for long-term development in patient safety in organisations. It is vital for patient safety that the planned changes related to the reporting will be documented and implemented more visibly. As a result, it will boost the confidence in managers’ work and strengthens all staff’s commitment to patient safety initiatives in an organisation.

**WHAT IS ALREADY KNOWN ON THIS TOPIC**

- Incident reporting systems (IRSs) are an established practice in hospitals and source of learning for an organisation.
- Planned improvement actions as responses to incidents are a crucial but less studied contributor to sustainable safety practices in hospitals.

**WHAT THIS STUDY ADDS**

- The improvement actions were most often documented in incidents related to medication and transfusion treatment, and the incidents related to patient accidents.
- Almost in all incident groups planned improvement actions were targeted on improving staff behaviour.
- Improvement actions associated with patient accidents were mostly focused on that particular patient’s further care.

**HOW THIS STUDY MIGHT AFFECT RESEARCH, PRACTICE OR POLICY**

- Patient safety incidents-related improvement actions serve for the long-term development of safety in an organisation.
- There is a need for extra structured documentation in IRSs regarding planned improvement actions and their implementation.
- More research is needed on selecting optimal improvement actions for different patient safety incidents and near misses.

## Introduction

During the last 15 years, incident reporting systems (IRSs) have been established in hospitals for identifying patient safety problems.^1 2^ IRSs are recognised as a system approach to detect, review and analyse safety incidents. They are used to evaluate the gained information and to implement the changes based on the planned improvement actions.^3^ Thus, IRSs have been used as tools to detect and manage risks,^4 5^ and raise staff’s awareness about patient safety.^6^

Even though IRSs have been established in hospitals globally,^1 2^ their implementation is variable.^1 7 8^ In Estonia, an initiative of patient safety has emerged as a focus in healthcare and society since the last decade, and therefore, is a relatively new concept. Hospitals are obligated to document adverse events such as transfusion reactions, postoperative wound infections, pressure ulcers, patient fall and transfer-related incidents with an outcome of patient harm, and adverse effects that occurred during the use of medicinal products.^9^ In addition, hospitals are advised to develop patient safety culture and implement IRSs.^10^ Hospitals have an autonomy to develop and use their own systems for incident reporting.^9^ In Estonia, with 1.331 million inhabitants and approximately 193 200 hospitalisations per year,^11^ 2 hospitals have implemented IRSs namely, the Tartu University Hospital, the only university hospital in the country since 2007, and the Tallinn Children’s Hospital since 2013. Original practical version of IRS was created based on the Finnish HaiPro (2006) reporting system^12^ and nowadays both hospitals have electronic versions. So far, the incidents have been reviewed and analysed on unit level and unit managers have been responsible for handling them in cooperation with other units and specialists. Till date, there is no national IRS in Estonia.

Despite of the advantages of IRSs, they have found to be underused,^3 6 7 13^ and especially planned improvement actions have been little focused.^14^ Although, incidences and planned improvement actions are stored in systems, their association with each other is poorly documented.^7 14^ It is crucial that planned improvement actions respond to the type of incidents, the level of harm and recognise patients’ individual characteristics. Implemented improvement actions at clinical level are usually focused on designing policies and procedures for care, changing the environments, advancing technical systems and developing and training care teams. Effective improvements have to be planned based on system insight and with systemic measures.^2^ On clinical level usually unit managers are responsible for dealing with reports analysis and designing improvement actions.^14^ However, they are found with short of time^6 15^ and trained experts to support them and carry out these actions.^7 8^ In addition, there is little knowledge on which improvement actions to be used for certain incidences^14^ and thus, challenges in assessing the appropriateness of planned improvement actions still remain.^6 16^ This knowledge is crucial to support the sustainable practices of patient safety in order to prevent incidents in the future, and ensure the long-term development of patient safety in organisations.^15^

Aim of this study was to describe and analyse associations of incidents and their improvement actions in hospital settings. This knowledge can be used by healthcare organisations to establish strategies and protocols for planning incidents-based improvement actions for patient safety. The research questions we addressed were (1) which incidents and planned improvement actions have been documented in the systems and (2) how incidents are associated with planned improvements?

## Methods

### Study design

We performed a document analysis^17^ of the incident reports collected in the two Estonian hospitals during the years 2018 and 2019. The method was chosen to provide a retrospective and systematic knowledge of the reported incidents based on the documents.^17 18^ Data were analysed by descriptive statistics, Pearson’s χ^2^ test or Fisher’s exact test, whenever applicable, and by logistic regression.

### Research settings

We conducted the study at Tartu University Hospital and Tallinn Children’s Hospital. These hospitals have been so far the only hospitals (out of 19 publicly funded hospitals), which implemented electronic IRSs in Estonia. Tartu University Hospital has approximately 950 beds, while Tallinn Children’s Hospital is with almost 150 beds, covering roughly 20% of all hospital beds in Estonia. Both hospitals are regional hospitals covering large-scale treatment specialties including acute psychiatric care for children and adults.

The IRSs in both hospitals were used for all units. The staff was educated on incident reporting. The reporting was voluntary, anonymous and the staff member could make an announcement whether they were witness, or detected, or themselves was a trigger of the incident. Unit level managers were responsible for reviewing and analysing the reports and planning improvements. The IRS was consisted of two main parts; the first part contained information from a staff member, and the second part was documented by the unit manager on the planned improvement actions for the incident.

### Patient and public involvement

Patients or the public were not involved in the design, conduct or reporting of our study.

### Data collection

Based on the WHO conceptual framework for patient safety^2 19^ and previously published literature,^1 7 8^ we developed a data extraction form for data collection. The extraction form was consisted of three parts. The first part (10 items) was focused on the characteristics of incident and the people involved; the second part (four items) was targeted on incident reporting and the reporter’s details and mitigating factors implemented for preventing more serious outcome of the incident. The third part (seven items) was focused on direct responding with ameliorating actions, the incident analysis and planning for improvement actions. We used 5% of data (2.5% from each hospital) as a pilot to test the extraction form to identify the items that can be detected based on the reports. As a result, we removed eight items, which were very difficult to extract from datasets (such as, incident reviewing and analysis part), and the final number of items was 7 for the first part, 2 for the second part and 4 for the third part.

After obtaining ethical permission, the researcher (EU) contacted the data specialists in the hospitals, who conducted data selection and anonymisation. The data of all the incidents documented during the period 1 January 2018–31 December 2019 was provided to the researcher in Excel spreadsheets (Microsoft Office, Washington, USA). The researcher extracted the targeted information from Excel into extraction forms, formalised in the web-based platform REDCap (Research Electronic Data Capture). During this phase, one report was removed because it was confirmed as a technical test report by the hospital.

### Data analysis

As the first phase of data analysis, all the data from incident reports at both hospitals were collected into REDCap and considered as one data set. This is because the two hospitals used different categorisations for the incident types, level of patient harm and planned improvement actions, which we recategorised according to the WHO framework for IRS.^19^ According to the recategorisation, we distinguished 14 items as main incident types, 14 items as planned improvement actions and 5 items as levels of harm. For statistical analysis, we condensed the incident types into six and planned improvement actions into four nominal variables (groups). In addition, we categorised the level of harm into four variables by summarising the categories as severe outcomes and death.

In both hospitals, most incident characteristics including contributing factors and improvement actions as responses to incidents were described in free text format in IRSs. Based on the text, we created new nominal variables for incident characteristics as ‘patient age’ and ‘incident time’ (table 1). Also, detection of incident, outcome for the organisation and contributing, mitigating and ameliorating factors as well as recommendations made by the reporter were categorised. In this paper, we will report incident types, level of harm and planned improvement actions.

**Table 1:** Characteristics of the incidents and presence or absence of planned improvement actions with differences between the groups

Characteristics	Planned improvement actions			Actions per case			
	Total	Yes	No	mean	OR	95% CI	P value
	n	n (%)					
Incidents groups based on incident type	1973	1643 (83)	330 (17)				<0.001*
Clinical processes and related care	561	463 (83)	98 (17)		0.10	0.74 to 1.35	0.988†
Clinical process/procedure	297	238 (80)	59 (20)	1.4			
Documentation	165	147 (89)	18 (11)	1.1			
Clinical administration	52	38 (73)	14 (27)	1.1			
Healthcare-associated infections	31	30 (97)	1 (3)	1.6			
Nutrition	16	10 (63)	6 (37)	1.4			
Medications and transfusion treatment	107	99 (93)	8 (7)		2.61	1.23 to 5.53	**0.012**†
Medication/intravenous fluids	86	81 (94)	5 (6)	1.3			
Blood/blood products	21	18 (86)	3 (14)	1			
Equipment							
Medical device/equipment	50	43 (86)	7 (14)	1.2	1.30	0.57 to 2.96	0.537†
People’s behaviour	608	502 (83)	106 (17)		1(ref)		
Behaviour (patient)	587	484 (82)	103 (18)	1.8			
Behaviour (relatives and other)	21	18 (86)	3 (14)	1.9			
Accidents with patients							
Patient accident	379	336 (89)	43 (11)	1.9	1.65	1.13 to 2.41	**0.010**†
Work organisation and environment	268	200 (75)	68 (25)		0.62	0.44 to 0.88	**0.007**†
Resources management	152	109 (72)	43 (28)	1.2			
Staff behaviour	63	44 (70)	19 (30)	1.1			
Infrastructure/building/fixtures	53	47 (89)	6 (11)	1.2			
Level of patient harm	1973	1643 (83)	330 (17)				0.001*
None	782	638 (82)	144 (18)	1(ref)			
Mild	585	483 (83)	102 (17)	1.07		0.81 to 1.41	0.641†
Moderate	561	491 (88)	70 (12)	1.58		1.16 to 2.16	**0.004**†
Severe/death	45	31 (69)	14 (31)	0.50		0.26 to 0.96	**0.038**†
Patient age	346	308 (89)	38 (11)				**0.002**‡
Newborn/infant (1 day to 1 year)	29	24 (83)	5 (17)	0.44		0.15 to 1.28	0.132†
Child/adolescent (1<+20)	249	228 (92)	21 (8)	1(ref)			
Adult (20<+65)	21	16 (76)	5 (24)	0.29		0.10 to 0.88	**0.029**†
Elderly (65<)	47	40 (85)	7 (15)	0.53		0.21 to 1.32	0.171†
Incident time	323	265 (82)	58 (18)				0.839*
Morning	94	77 (82)	17 (18)				
Afternoon	85	71 (84)	14 (16)				
Evening	80	67 (84)	13 (16)				
Night	64	50 (78)	14 (22)				

*Pearson’s χ2 test.

†Logistic regression model.

‡Fisher’s exact test.

!, Bold figures indicate statistically significant values.

For the statistical analysis, we exported the data to R V.4.1.2 (The R Foundation for Statistical Computing, R Core Team). Descriptive statistics was used to summarise a distribution of incident types and level of harm. For identifying differences between the groups, we formed a binary variable of planned improvement actions—if the actions were documented or not. We assessed an association between the documentation of improvement actions and background characteristics of incidents using Pearson’s χ^2^ test or Fisher’s exact test, whenever applicable. A p value of 0.05 was selected as a cut-off for significance. ORs with corresponding 95% CIs and p values were calculated using logistic regression models to further examine whether planned improvement actions were documented or not in a system for six categories of incident groups, four variables of patient harm and four variables of patient age.

## Results

### Reported incidents and planned improvement actions

**Figure 1:**
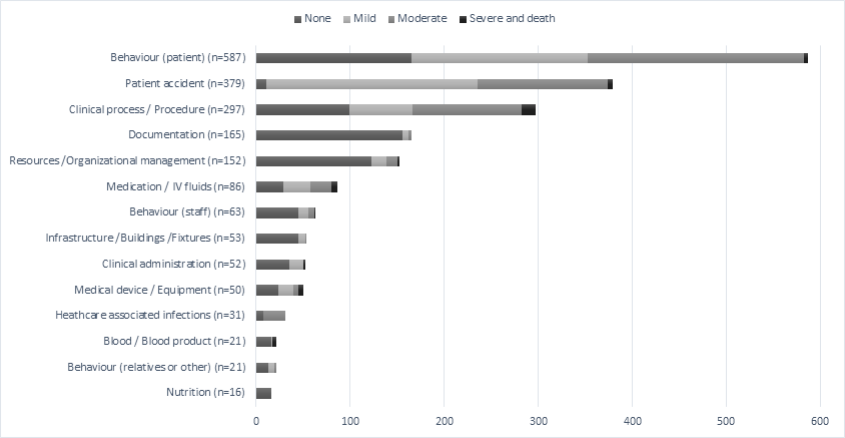
A distribution of the reported incidents and a level of harm in the main types of incidents (N=1973) (2018–2019).

In total, 1973 incidents were reported during the 2-year period in both regional hospitals (table 1). The most commonly reported incidents were about patients’ violent or self-harming behaviour, and patient accidents such as falls. The third most often reported incidents were about clinical processes and procedures such as a procedure was not performed or was performed to a wrong patient. This was followed by the incidents related to documentation and resources management (figure 1).

Out of all the reported incidents, more than one-third were non-harm incidents (n=782), less than one-third were of mild-harm (n=585) and moderate harm incidents each (n=561) (table 1). Mild-harm and moderate harm incidents together formed approximately two-thirds of all patient behaviour-related incidents (n=187, n=230). The same harm levels were on the top in patient accident-related incidents (n=224, n=139). Incidents with outcomes of severe harm (n=41) and death (n=4) were the most among (n=15) the incidents related to clinical processes and procedures (figure 1).

Improvement actions were planned for 83% of all the reported incidents (table 1). The highest average number (mean (x̄), min=1, max=4) of planned improvement actions per incident were calculated for the patient accident-related incidents, the incidents related to patients’ relatives or other people’s behaviour and the incidents related to patient behaviour.

**Figure 2:**
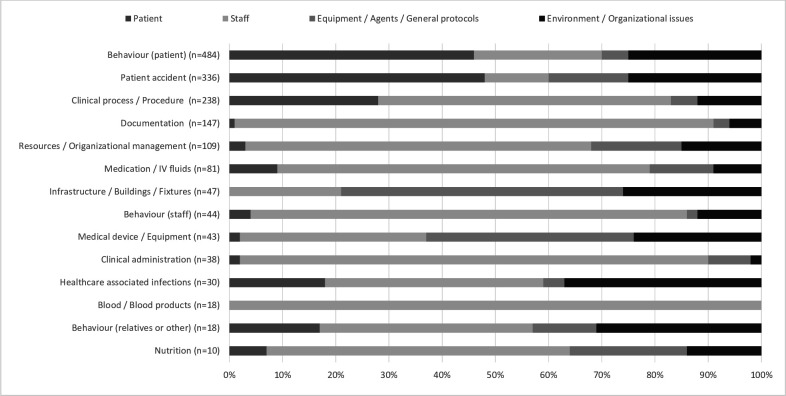
A distribution of planned improvement actions in different incident types (2018–2019).

Planned and documented improvement actions were focused on direct patient care, staff, equipment and general protocols, and environmental and organisational issues (figure 2). A proportion of staff focused improvements was the highest among the eight incident types as demonstrated in figure 2.

Improvement actions planned for direct patient care were consisted of an improved support and care for the involved patient, patient education or counselling, and provision of extra equipment for better monitoring (table 2). Such recommendations were mostly proposed for the incidents related to patient accidents like falls and patient self-harming or aggressive behaviour (figure 2). Actions targeting the staff were consisted of providing support to the involved staff, mostly through discussions after patient harm incidents and a supervision and training to the involved teams. Also, the improvement actions were planned for developing new protocols and updating the existing ones, assuring the availability of guidelines and protocols, reminding and controlling the adherence to them, and assuring adequate staffing in terms of staff number and quality in case understaffing as the cause of the incident (table 2).

**Table 2:** Planned improvement actions with exemplar quotes from the incident reports

Planned improvement actions	n(%)	Example quotes
Patient care focused	822 (32)	
Adequate support and care to patient		Placing a patient into a private room to calm down Managing a challenging behaviour with a ‘Verge method’
Patient education/counselling/decision support		Educating a patient to handle irritating situations Counselling a patient for agreeing to treatment Talking to patient, relatives, fellow patients
Monitoring equipment/medication dispensing aids to patient		Providing a dispensing aid for medicines Organising an extra monitor Providing additional aids to support patient’s mobility
Staff focused	932 (37)	
Staff orientation/supervision/assistance		Discussion of an incident analysis with involved staff members Supervising the team
Staff training		Special training was planned for taking lab tests
Checklists/protocols/policies availability and adherence		Reminding about an adherence to the guideline New protocol needed
Adequate staff numbers/quality		Calling out for extra staff/a security specialist
Equipment/agents/general protocols	260 (10)	
Provision/improvement of resources/equipment/medication alerts/systems/services		Replacing faulty instruments Using stop sign stickers for additional attention Informing/calling for Police
General protocols and guidelines support		Organising a patient transport in general level Organising an incident-related discussion between partner institutions
Environment/organisational issues	520 (21)	
Environmental changes/matching physical environment		Contact isolation established in the ward Furnishing a special room (for an aggressive patient)
Organising risk assessment/root cause analysis/audits		Organising a risk analysis Informing a medical company about faulty equipment
Improving leadership/guidance		Suggesting changes in working organisation
Matching staff/planning adequate staff		Hiring a security service
Improving a safety culture		Reminding about IRS
Total	2534 (100)	

IRS, incident reporting system.

All the incidents related to blood or blood products, and 90% of related to documentation, had planned improvement actions focusing on staff. Mostly those were proposing reminding and training for better adherence to the guidelines. ‘Staff training’ as an improvement action was mentioned often but was not described in detail. Some reports had additional information indicating on the training topic (‘taking lab tests’, ‘administration of medicines’), the target group (nurses and nursing aides, only nurses), organisation (in cooperation with the pharmacy department), and the time when the training would be or was held. Staff related actions were modestly indicated in the incidents related to patient accidents (12% of all such incidents) (figure 2).

Equipment and general protocols-related improvement actions were about providing extra resources, such as an equipment, new systems and services, also general level protocols and support (table 2). Mostly such recommendations were made in case of the incidents related to infrastructure (53%) and medical devices (39%) (figure 2). Environment and organisational issues-related improvement actions were targeted changing or matching physical environment or staff resources, arranging access to protocols and decision support. Improving leadership, guidance and safety culture, as well as organising audits and risk assessments, including root cause analysis were also among the documented improvement actions (table 2), but rarely described in detail. Such actions were usually planned for the incidents related to healthcare associated infections (37%) and relatives or other persons’ behaviour (31%) (figure 2).

### Associations between incidents characteristics and planned improvement actions

There were significant differences between the planned improvement actions and the incident types, level of harm and patient age. No significant differences were found for incident time (table 1). Based on the incident type, medications and transfusion treatment-related incidents (OR=2.61) and patient accident-related incidents (OR=1.65) got planned improvement actions significantly more in numbers as compared with the incidents regarding people’s behaviour. Significantly lower odds (1.6 times lower in numbers) were found for the incidents related to work organisation and environment (table 1).

In case of the level of patient harm, the odds for documented planned improvement actions were 58% higher for the incidents with moderate harm and twice lower (OR=0.50) for the incidents with severe harm or death when compared with the incidents with no harm. Regarding patient age, the incidents with adults had three times lower odds for improvement actions (OR=0.29) than the incidents with children/adolescents. (table 1).

## Discussion

Based on our findings, the reported patient safety incidents were mostly related to patients’ violent or self-harming behaviour. They were caused by patients’ mental state or their disagreements on treatments and hospital rules. The second most common reported incidents were patient falls, followed by the incidents related to delayed, omitted or wrong clinical procedures. The most often planned improvement actions were focused on patient care with education and monitoring or on staff supervision and training. Regarding the associations, the most often improvement actions were focused on medication and transfusion-related incidents and were targeted improving staff behaviour. Second most frequent improvement actions were focused on patient accidents and providing direct care to the patient. Planned improvement actions were most commonly documented for the incidents with moderate harm, and more frequently involving children and adolescents than adults.

Our results are contradictory in relation to previous register-based studies. They are in line with the studies, where the most often reported incidences were about patient falls^14 20^ and clinical procedures.^7 21^ This can be partly explained by the nurses’ close role in patient monitoring^20^ and also as a consequence of staffing arrangements.^13 20^ Further, it is important to note that in Estonia it is obligatory for hospitals to document patient falls resulting in harm since 2014.^9^ Based on previous studies reporting of incidents correlates with legislation^4 8 16^ and internationally has been advised by WHO.^15^ Thus, in future, it could be beneficial to consider national policy in Estonia to identify other focuses of regulation to support patient safety. In the current study, the proportion of incidents related to medications were less reported as compared with the previously published studies.^1 13 22–24^ It is noteworthy that there is no previous information on the level and reasons for medication incidents in Estonia. In the previous studies, the low level of medication-related incident reporting was explained as a lack of clear definitions of medication error,^25 26^ a lack of feedback on reporting^3 8 26^ and a reporter’s previous experience with such kind of incidents.^22^ In addition, a fear of blaming was noticed as a reason for not reporting medication incidents.^22 26^ Therefore, there is a need to identify the reasons for low level medication incidents reporting in Estonia and to consider, if there is a need to develop a blame free patient safety culture^6 25^ and relevant patient safety educational interventions for healthcare staff.^1 7 8^

In the current study, the results regarding the type and prevalence of improvement actions, and their association to the incident types are in line with previous studies.^1 8 14^ The advantage is that they strongly reflect a patient-focused and individuals behaviour targeted approach.^6^ However, individual cases centred improvements have been found to have a limited power^3 16 27^ as compared with system-focused improvements.^15 28^ In this study, we also found a low level of improvement actions focusing on the organisation and environment. This is in line with a previous study, where organisation-level actions were seen to be too complex due to coordination problems impeding their implementation.^6^ According to the WHO guidelines,^19^ the permanent changes should be achieved by actions at the organisational level. Moreover, data aggregation from individual case analyses and their investigation will reveal insights of hidden risks and errors that can be solved by system changes to achieve benefits in larger context.^27^ Hospitals need to consider and accept that the system-level actions require to establish intraorganisation collaboration to identify, share and evaluate protocols for planning improvement actions as a part of strategical management of patient safety.^3 27^

Our study provided new knowledge that the incidents with moderate level of harm were associated with higher number of improvement actions. In the future, more attention needs to be paid also to improvement actions for no-harm incidents and near misses. This would avoid further recurrence of incidents and hence is an essential step for the long-term prevention of incidences.^28^ Thus, to achieve permanent improvements, a more system-level approach for developing improvement action should be used. Therefore, IRSs implementation regarding improvement actions planning and documentation needs more in-depth research, for developing better strategies and protocols for it. As a result, confidence among staff and managers for IRSs utilisation will advance and patient safety would be supported in organisations.

The strength of our study is its large sample size; we reviewed a full sample of the incident reports documented over the period of 2 years in the two hospitals of Estonia. This ensured an enclouser of all time trends or seasonal impacts that might have alleviated the content and frequency of reporting. As both hospitals had electronic systems in use for already some years, the probable difficulties in the implementation of IRS were overcome. Reliability of the data collection process and analysis was ensured by the use of a structured data extraction form. Still, as data extraction and coding of incident report contents were predominately undertaken by a single author (EU), subjective interpretations of the information within reports may exist. Nonetheless, this study has managed to provide an insight of the reporting practices in hospitals using electronic IRSs. Moreover, it gives a valuable overview of planned improvement actions documented in systems and their associations with the background characteristics of the incidents. However, it is yet to be evaluated, if the current structure of IRSs responds to updated recommendations of a patient involvement in incident investigation.^29^ In our dataset, we found only a few reports where patients’ or their relatives’ involvement in reviewing the incidents was documented.

The method of document analysis has a few limitations linked to retrospective data collection and data quality.^17 18^ First, the incident reports were originally created for other purposes than research; therefore, in some cases, the data was insufficient, too fragmented or even missing. Second, the original data extracted from the hospitals’ systems had some differences in terms of the data reporting practices, and some details were not provided to the researchers for the analysis. Third, in case of one hospital, the planned improvement actions documented in original datasets were often in the form of predefined categories lacking a descriptive content of the actions (like, what really was decided to improve or change). In the other hospital although the planned improvements were available in the form of free text descriptions, the structure and consistency of the actions were lacking.

## Conclusion

Based on our study, we can conclude that patient safety incidents and their associations to improvement actions need more attention in future. Our study findings on improvement actions focusing on individual patient care events, reflect a patient-focused approach when replying to people’s behaviour. The detected associations regarding medication and transfusion-related incidents mirror an elevated focus on improving and influencing staff’s behaviour, not so much changing the conditions they work in. The low level of organisation and environment-related improvement actions can be seen as a risk for long-term development in patient safety in organisation. However, IRSs can be more effectively used for patient safety developments when managers and staff will provide better input with precise documentation, and organisation would support patient involvement in revising incidents. Moreover, this can create opportunities for implementing improvement actions and studying their effects; these records can promote responding to incidents, and implemented changes and their benefits can be better visible in practice. Consequentially, it will boost the confidence in managers, and their commitment to patient safety initiatives in the organisations will be better supported. In future, more knowledge is needed about optimal improvement actions for different patient safety incidences and near misses. This knowledge would support organisations to develop structures and strategies for patient safety.

## Data Availability

Data are available on reasonable request. The data are not available publicly. However, they are available on a reasonable request to the corresponding author and with permission from involved hospitals.
